# Retracted: Medical Dataset Classification: A Machine Learning Paradigm Integrating Particle Swarm Optimization with Extreme Learning Machine Classifier

**DOI:** 10.1155/2016/7137054

**Published:** 2016-04-21

**Authors:** The Scientific World Journal

The Scientific World Journal has retracted the article titled “Medical Dataset Classification: A Machine Learning Paradigm Integrating Particle Swarm Optimization with Extreme Learning Machine Classifier” [1]. After conducting a thorough investigation, we have strong reason to believe that the peer review process was compromised.

This article was originally submitted to a Special Issue titled “Recent Advances in Metaheuristics and its Hybrids.” In late 2015, Dr. Xavier Delorme, the lead guest editor on the Special Issue, alerted us that his identity had been compromised. After further investigation, we discovered that several peer review reports in this issue had been submitted from similarly compromised email accounts.

We are retracting the articles in keeping with the “COPE statement on inappropriate manipulation of the peer review process.” There is no evidence that any of the authors or editors, including Dr. Delorme, were aware of this misconduct.
